# Fatigue in intensive care survivors one year after discharge

**DOI:** 10.1186/s12955-016-0554-z

**Published:** 2016-10-18

**Authors:** Savino Spadaro, Maurizia Capuzzo, Giorgia Valpiani, Sara Bertacchini, Riccardo Ragazzi, Francesca Dalla Corte, Simona Terranova, Elisabetta Marangoni, Carlo Alberto Volta

**Affiliations:** 1 Department of Morphology, Experimental Medicine and Surgery, Section of Anaesthesia and Intensive Care, University of Ferrara, Azienda Ospedaliero-Universitaria S. Anna, Via Aldo Moro, 8. 44121, Cona, Ferrara Italy; 2 Research and Innovation Office, Azienda Ospedaliero-Universitaria S. Anna, Cona, Ferrara Italy

**Keywords:** Outcome, Fatigue, FACIT-F scale, Quality of life, Intensive care

## Abstract

**Background:**

Fatigue has not been investigated in long-term Intensive Care Unit (ICU) survivors. This study aimed to assess fatigue through a specific instrument, namely the Functional Assessment of Chronic Illness Therapy Fatigue (FACIT-F) scale, in ICU survivors one year after hospital discharge. A secondary aim was to compare the findings of FACIT-F with those of the Vitality domain (VT) of the 36-item Short-Form Health Survey (SF-36).

**Methods:**

This prospective cohort study was performed on 56 adult patients with a Length Of Stay (LOS) in ICU longer than 72 h. At one year after hospital discharge, FACIT-F and SF-36 questionnaires were administered to consenting patients by direct interview. FACIT-F was measured as raw (range 0–52), and FACIT-F-trans value (range 0–100). Past medical history, and demographic and clinical ICU-related variables were collected.

**Results:**

The patients’ median age was 67.5, Simplified Acute Physiology Score II 31, and LOS in ICU 5 days. The median raw FACIT-F of the patients was 41, and Cronbach’s α was 0.937. The correlation coefficient between FACIT-F-trans and VT of SF-36 was 0.660 (*p* < 0.001). Both FACIT-F and VT were related to dyspnoea scale (*p* = 0.01). A Bland-Altman plot of VT vs FACIT-F-trans showed a bias of –0.8 with 95 % limits of agreement from 35.7 to –34.1. The linear regression between differences and means was 0.639, suggesting a significant proportional bias.

**Conclusions:**

The 13-item FACIT-F questionnaire is valid to assess fatigue of long-term ICU survivors. VT of SF-36 relates to FACIT-F, but consists of only four items assessing two positive and two negative aspects. FACIT-F grasps the negative aspects of fatigue better than VT. Specific tools assess specific conditions better that general tools.

**Trial registration:**

ClinicalTrials.gov: NCT02684877.

## Background

Fatigue, perceived by the individual as an overwhelming sense of tiredness at rest, is one of the symptoms most commonly reported by patients [1]. There are many causes of pathological fatigue, including both neurological and non-neurological diseases. Within the last group, cancer, infections, and drugs are factors associated with fatigue [1]. For many cancer patients, fatigue is the most distressing untreated symptom that causes the greatest amount of interference with daily life [2]. Cancer Related Fatigue affects quality of life adversely by reducing mental and physical functioning, disturbing mood, and interfering with usual activities [3]. A survey on Australian and Canadian haematological cancer survivors identified ‘Dealing with feeling tired’ as the highest concern of survivors [4]. Interestingly, fatigue continues to be a problem for breast carcinoma disease-free survivors, 5–10 years after the diagnosis, especially for women treated with both radiation and chemotherapy [5].

Some evidence suggest that patients who have been critically ill continue to face a multitude of physical, psychological, and social difficulties in the long term after discharge [6]. Fatigue has not been widely explored in Intensive Care Unit (ICU) patients but more than 50 % of ICU survivors reported lowered energy levels and fatigue in the first year after discharge [7]. More recently, an extensive survey on long-term complications reported by the General Practitioners who followed ICU survivors at one year showed that decreased exercise tolerance and chronic fatigue were among those most often reported [8], underlying the clinical relevance of chronic fatigue in ICU survivors. However, no study has specifically assessed fatigue among long-term ICU survivors through a validated instrument.

Many instruments to measure fatigue have been proposed in rheumatic conditions [9]. One of the most used is the Functional Assessment Chronic Illness Therapy-Fatigue (FACIT-F) scale. It was developed in 1997 to measure fatigue in oncology patients with anaemia [10], and has been validated in a sample of the general USA population [11], and in patients with rheumatic diseases [9, 12]. Recently, FACIT-F has been validated in patients with inflammatory bowel disease [13], Chronic Obstructive Pulmonary Disease (COPD) [14, 15] and iron deficiency anaemia [16], resulting in one of the most appropriate fatigue questionnaires [17].

Many studies on validation of fatigue questionnaires analysed the correlation between the FACIT-F and the Vitality domain (VT) of the general 36-item Short-Form Health Survey (SF-36) [9]. A strong correlation was found between FACIT-F scale and VT of SF-36, though FACIT-F items cover a wider range of fatigue than the VT items of SF-36 [9, 16].

The aim of the present study was to measure fatigue through a specific instrument (FACIT-F) administered to ICU survivors one year after discharge from the hospital. A secondary aim was to compare the findings of fatigue assessed by FACIT-F with those obtained by the VT of SF-36.

## Methods

The study was conducted in a mixed medical-surgical 6-bed ICU of a university hospital of 710 beds located in the Northeast of Italy. This ICU serves all thoracic, vascular, and high-risk abdominal surgery patients and about half of the medical ward patients of the hospital.

All consecutive adult ICU patients discharged between March 2013 and October 2014 were considered for enrolment. We included patients aged more than 18 years, staying in ICU for at least 72 h, without pre-existing cognitive dysfunction or language barrier. One year after hospital discharge, we assessed the vital status of the patients through the Vital Statistics Offices. We excluded patients residing farther than 30 km from the hospital, and we contacted the ICU survivors by phone to ask them to participate in the study. Those who agreed received an appointment at the hospital for a date and time convenient for them.

The demographics and clinical data of the study patients were retrieved from the ICU database. The software for this (PROSAFE) is made available by the Italian GiViTI group, and has been used in the study ICU since 2008 for daily collection and storage of patient data. For each participant, the following information was retrieved: gender, age, Body Mass Index (BMI), pre-existing comorbidities (like hypertension, COPD, cardiac failure of NYHA class III or IV, and diabetes), reason for ICU admission, type of admission (elective surgery, emergency surgery, or medical admission), date of hospital and ICU admission, presence of severe sepsis or septic shock (at ICU admission or during ICU stay), main treatments received (administration of vasoactive amines, mechanical ventilation, renal replacement techniques), date of ICU and hospital discharge and vital status at hospital discharge. The following computed data were also retrieved: Simplified Acute Physiology Score (SAPS) II [18] and Sequential Organ Failure Assessment (SOFA) Score [19] which referred to the first 24 h in ICU, and Length Of Stay (LOS) referring to the number of days in ICU and in hospital after ICU discharge.

### Instruments

At the time of the appointment at the hospital, a researcher not involved in the care of the patient during his/her stay in ICU welcomed the patient, obtained signed consent, and administered the following questionnaires in a calm and confidential environment. The time scheduled for each appointment was 30 min and it was generally observed.

The *Functional Assessment of Chronic Illness Therapy for Fatigue* (FACIT-F) scale is a sub-scale of a general questionnaire developed to assess anaemia-related symptoms in cancer patients [10]. FACIT-F consists of 13 items referring to the previous seven days. Each item allows five response options from ‘Not at all’ (scored 4) to ‘Very much’ (scored 0) with two items needing a reverse score. The scores are summed, multiplied by 13, then divided by the number of items actually answered, thereby allowing calculation for missing items. The final (raw) score ranges from 0 to 52, with higher scores representing less fatigue, and lower scores more fatigue. The raw scores can be transformed into interval measures (FACIT-F-trans) ranging from 0 to 100, according to an interval metric proposed by Cella et al. [11].

The *Short-Form 36* (SF-36 version 1) [20] was administered in the previously validated Italian version [21]. The questionnaire refers to the four previous weeks, and consists of eight multi-item dimensions, i.e., physical functioning (PF), role limitation due to physical problems (RP), bodily pain (BP), general health (GH), vitality (VT), social functioning (SF), role limitation due to emotional problems (RE), and mental health (MH). Scores with a range from 0 to 100 are obtained for each dimension. Higher scores represent better functioning. Differences in SF-36 scores of more than 5 points were reported as clinically meaningful [22].

The *Medical Research Council Dyspnoea (MRCD) Scale* is suitable to assess the extent to which breathlessness affects patient mobility during daily activities [23]. It consists of five statements about perceived breathlessness, from grade 1 (breathless with strenuous exercise) to grade 5 (too breathless to leave the house). We administered the MRCD Scale to check for a correlation between this scale and both FACIT-F and VT of SF-36. We predicted that FACIT-F scores would be significantly worse (lower) in patients with mobility affected by breathlessness than in those without it. This analysis was performed to demonstrate the construct validity of both FACIT-F and VT.

The Ethics Committee of the Province of Ferrara approved the study protocol (n. 140696), and required the written consent of each participant. We received permission for the appointed institution to use the FACIT-F and SF-36 questionnaires.

### Statistics

Categorical data are presented as count (%). The Shapiro-Wilk test was used to assess whether continuous variables were normally distributed, and data are shown as mean (±SD), or median with 1st and 3rd quartiles [Q1-Q3], accordingly. A Chi squared test, or a Fisher’s exact test when appropriate, was used for categorical comparisons, and a Mann-Whitney *U* test for comparisons of non-normally distributed variables. We investigated the internal consistency of the FACIT-F questionnaire using Cronbach’s α coefficient. Spearman’s correlation coefficient was used to analyse correlations between FACIT-F and VT of SF-36, and between both FACIT-F and VT and the MRCD Scale. The Bland Altman plot was used to assess the agreement between FACIT-F-Trans and VT of SF-36 (both ranging 0-100). Linear regression between the differences and the means, which should be close to zero, was used to evaluate the presence of a proportional bias.

For each SF-36 dimension, we computed the normal values for a population matched to our study patients for gender and age [24], and we compared mean values of our study patients with the Italian adjusted normative population using an unpaired *t* test.

A Type I error in two-tailed tests was considered significant (α 0.05). Statistical analysis was performed using the software packages SPSS v. 11.5 (IBM, New York, USA) and STATA 12.1 (StataCorp, Texas, USA).

Due to the lack of studies specifically assessing fatigue in long-term ICU survivors, we relied on the raw values of FACIT-F (43.6 ± 9.4) for the general USA population [11]. To detect a 10 % difference (4.36) between the mean score of our patients and that of the general USA population (α = 0.05, power = 0.80), the necessary sample size was calculated as 37 patients. Considering that long-term mortality and dropouts could markedly decrease the number of the patients who can be assessed at one year, we decided that the time hypothetically requested for reaching the sample size was about 16 months (March 2013 - October 2014).

## Results

**Fig. 1:**
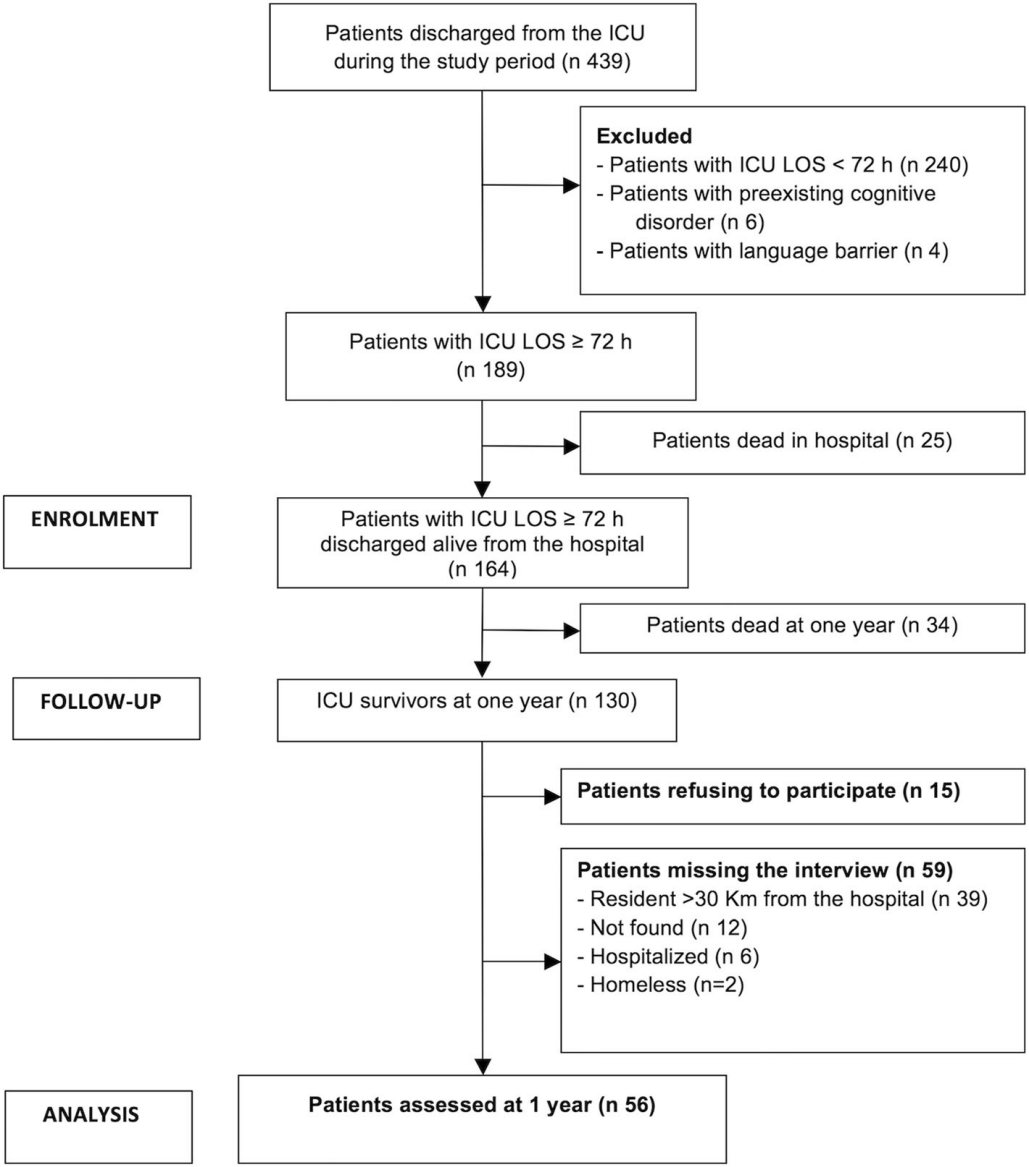
Flowchart of the study patients

During the study period, 439 patients were discharged from the ICU alive (Fig. 1). Two hundred and forty of them, who stayed in ICU less than 72 h, as well as 6 patients with a pre-existing cognitive disorder and 4 patients with language barriers, were excluded. Of the remaining 189 patients, 25 died in hospital after ICU discharge, and 34 were dead at one year according to the Vital Statistics Offices. Of the 130 patients alive one year after ICU discharge, 15 refused to participate in the study, and no information about them was collected. Thirty-nine patients who lived farther than 30 km from the hospital, 12 patients who were not found despite multiple attempts to contact them, six patients hospitalized at one year, and two homeless people could not be interviewed. At one year, we directly interviewed 56 patients.

The clinical characteristics of the study group and of the 59 patients who missed the interview (Fig. 1) are reported in Table 1. The two groups showed a statistically significant difference in the BMI and in the LOS in ICU. Of the patients assessed at one year (mean age 67.0 ± 10.6 y), only 25 % had no comorbidity. While 36 of the study admissions were surgical (64.3 %), with 24 urgent and 12 elective admissions, the other 20 admissions were medical (35.7 %). The most common reasons for ICU admission were acute respiratory failure (48) and severe sepsis or septic shock (15) amounting to 85.7 % and 26.8 % respectively. Forty-eight patients (85.7 %) received mechanical ventilation (mean duration 6.5 ± 9.8 days, range 1-54), and 13 (23.2 %) received vasoactive amines.

**Table 1:** Clinical characteristics of the patients interviewed and missing the interview (refusals excluded). Data are shown as median with quartiles [Q1-Q3]. Categorical data are reported as number with percentage in brackets

	Interviewed	Missing the interview	*p* value^*^
Patients, *n* (%)	56	59	
Gender male, *n* (%)	38 (67.8)	39 (66.1)	0.844
Age, years, median [Q1-Q3]	67.5 [59.0-74.0]	73 [62.7-77.0]	0.214
Body Mass Index, kg/m^2^, median [Q1-Q3]	26.8 [23.5-30.6]	24.7 [22.1-27.3]	0.018
Comorbidities, *n* (%)			
absent	14 (25.0)	18 (30.5)	0.798
one	25 (44.6)	25 (42.4)	
more than one	17 (30.4)	16 (27.1)	
Type of ICU admission, *n* (%)			
Medical	20 (35.7)	24 (40.7)	0.612
Urgent surgical	24 (42.9)	20 (33.9)	
Elective surgical	12 (21.4)	15 (25.4)	
Diagnostic group, *n* (%)			
Respiratory pathology	17 (30.4)	25 (42.4)	0.793
Cardiovascular pathology	9 (16.0)	8 (13.6)	
Gastrointestinal pathology	25 (44.6)	21 (35.6)	
Neurological pathology	3 (5.4)	1 (1.7)	
Trauma	1 (1.8)	1 (1.7)	
Other	1 (1.8)	2 (3.4)	
SAPS II score, median [Q1-Q3]	31 [27.0-37.7]	33 [26.0-48.2]	0.231
SOFA score, median [Q1-Q3]	4.0 [3.0-6.0]	4.0 [2.0-8.0]	0.389
Severe sepsis/septic shock, *n* (%)			
at ICU admission	15 (26.8)	16 (27.1)	0.568
during ICU stay	18 (32.1)	19 (32.2)	0.994
Treatments received in ICU			
Mechanical ventilation, n (%)	48 (85.7)	47 (79.7)	0.465
Duration of MV ^a^, days, median [Q1-Q3]	3 [1.2-9.0]	4 [2.0-10.2]	0.328
Vasoactive amines, *n* (%)	13 (23.2)	22 (37.3)	0.110
Renal Replacement Treatment, *n* (%)	1 (1.8)	1 (1.7)	0.970
LOS^b^ in ICU, days, median [Q1-Q3]	5 [3.0-12.0]	7 [5.0-12.2]	0.012
LOS^b^ in hospital, days, median [Q1-Q3]	10.5 [7.0-17.0]	8.5 [5.0-15.5]	0.103

^*^p value: statistical significance

^a^ *MV* mechanical ventilation

^b^ *LOS* length of stay

**Fig. 2:**
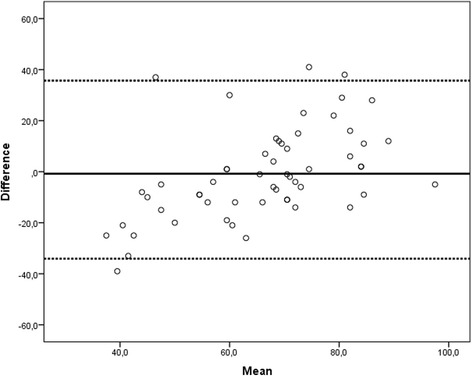
Bland-Altman plot to assess the agreement between FACIT-F-Trans and Vitality dimension (VT) of SF-36. Each marker represents one patient. The *x-*axis shows the mean value of the two assessments and the *y-*axis shows the difference between the two assessments. The *solid line* represents the overall mean difference, and the *dashed lines* represents the 95 % limits of agreement (1.96 SD mean difference). Where perfect agreement is observed, individual points line up along the 0 line of the y-axis

The mean raw FACIT-F score of the study patients was 39.1 (±10.1, median 41, Q1-Q3 34-47), and the FACIT-F-trans value was 66.4 (±12.4, median 66, Q1-Q3 59-74). Cronbach’s α of FACIT-F was 0.937. The correlation coefficient between the FACIT-F-trans and the VT of SF-36 was 0.660 (*p* < 0.001). The Bland Altman plot of FACIT-F-Trans vs VT showed a bias of -0.8 with 95 % limits of agreement from -34.1 to 35.7 (Fig. 2). The linear regression between differences and means was 0.639, suggesting a significant proportional bias.

**Fig. 3:**
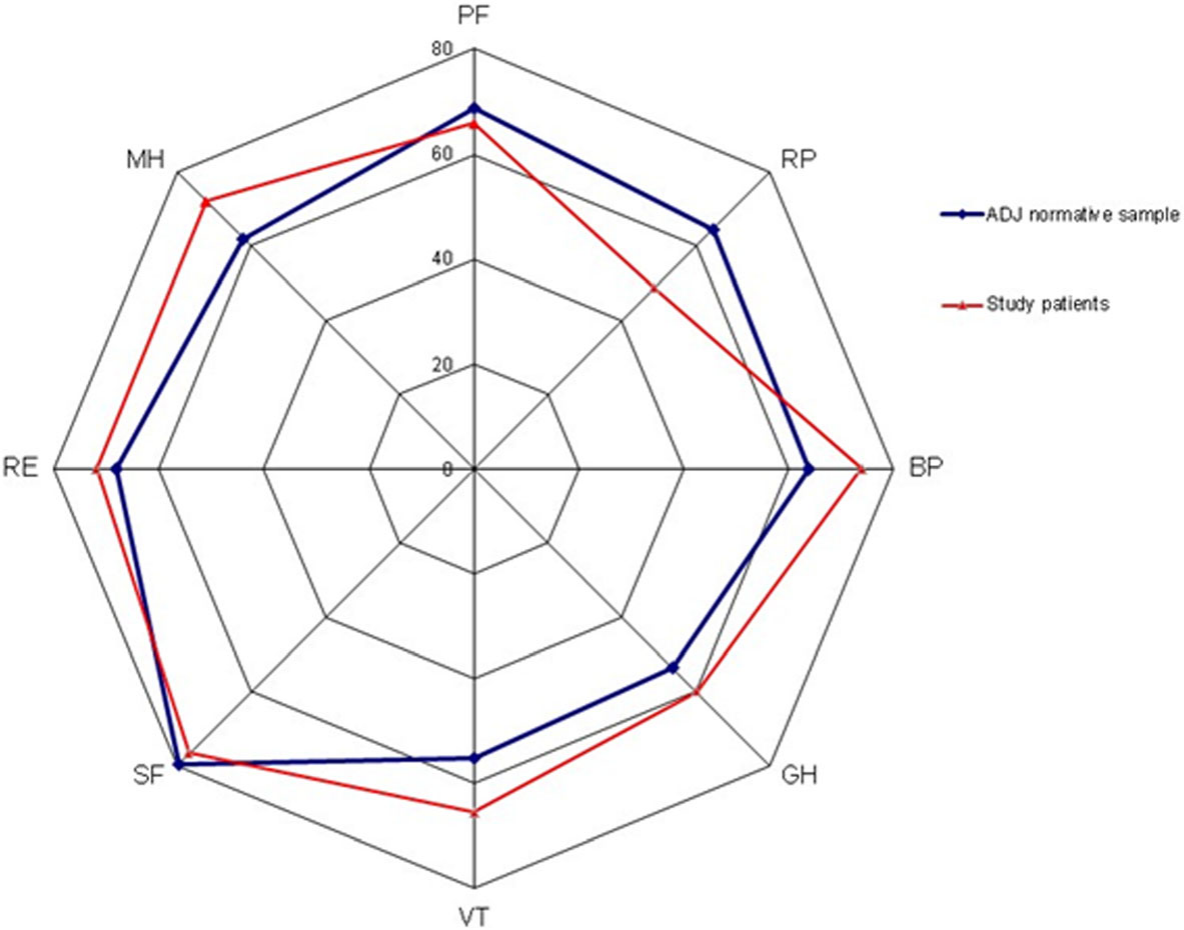
Comparison between the mean values of the dimensions of Short-Form 36 reported by the study patients and those of the Italian adjusted (ADJ) normative population using unpaired *t* test. PF: physical functioning; RP: role limitation due to physical problems; BP bodily pain; GH: general health; VT: vitality; SF: social functioning; RE: role limitation due to emotional problems; MH: mental health

The SF-36 data for the study group and the adjusted Italian population [24] are reported in Table 2. The radar chart for the study group and the adjusted Italian normal population is shown in Fig. 3.

**Table 2:** Short Form-36 data of the study patients shown as mean, Standard Deviation (SD), median and quartiles [Q1-Q3]. For each SF-36 dimension, the values for the study patients and the normative values are reported, with delta value (mean of study patients –normative mean). Negative delta values represent a quality of life of the study patients worse than that of the normal population

		Study patients	Normative values^a^	Delta	*p* value ^*^
SF-36^b^					
PF	mean	65.89	68.95	-3.1	0.510
	SD	29.45	18.21		
	Median	75			
	Q1-Q3	44-90			
RP	mean	48.66	64.61	-15.9**	0.006
	SD	39.14	15.47		
	Median	50			
	Q1-Q3	0-100			
BP	mean	73.98	63.95	+10	0.021
	SD	29.81	11.37		
	Median	84			
	Q1-Q3	52-100			
GH	mean	60.05	53.81	+6.2	0.074
	SD	23.19	11.38		
	Median	67			
	Q1-Q3	39-77			
VT	mean	65.63	55.34	+10.3	0.001
	SD	20.45	8.14		
	Median	68			
	Q1-Q3	50-80			
SF	mean	76.79	79.70	-2.9	0.435
	SD	26.91	6.61		
	Median	88			
	Q1-Q3	59-100			
RE	mean	72.02	68.06	-4.0	0.496
	SD	42.06	10.16		
	Median	100			
	Q1-Q3	58-100			
MH	mean	72.21	62.06	+10.1	0.001
	SD	20.14	5.92		
	Median	76			
	Q1-Q3	60-88			

^a^ Data of the Italian normative sample collected by Apolone et al. [24] adjusted for gender and age

^b^ *PF* physical functioning, *RP* role limitation due to physical problems, *BP* bodily pain, *GH* general health, *VT* vitality, *SF* social functioning, *RE* role limitation due to emotional problems, *MH* mental health (range 0-100 with higher scores represent better functioning)

^*^ *p* value: statistical significance according to unpaired *t* test

** Delta value showing a minimum clinically significant difference of more than 5 points, consistent with quality of life worse than the adjusted normal population

Eighteen patients (32 %) reported a perceived breathlessness grade of one (normal value) on the MRCD Scale. Of the remaining patients, 17 (30 %) were in the second grade, 10 (18 %) in the third, 8 (14 %) in the fourth and 3 (6 %) in the fifth grade of the MRCD Scale. The correlation coefficient between the MRCD Scale and FACIT-F-Trans was -0.593 (*p* = 0.01), and that between the MRCD Scale and VT was -0.430 (*p* = 0.01).

## Discussion

Chronic fatigue is distressing and greatly interferes with daily activities. It may result from depression but can also cause it, and however worsens the quality of life of patients and their families. Assessing fatigue by a validated instrument is important because it may allow physicians to take care of the patients with chronic fatigue and their families.

Our study patients reported a value of fatigue, assessed by the FACIT-F scale, not too far from that of the general USA population (median 41 vs 47) [11]. The FACIT-F-trans values were significantly related to the VT of SF-36 (Spearman’s rho 0.660). The VT dimension of SF-36 reported by these patients was better than that of the adjusted Italian population (mean 65.6 vs 55.3), both statistically (p = 0.001), and clinically (because it was higher than 5 points [22]). The Bland-Altman plot of SF-36 VT versus FACIT-F-trans showed a small bias (-0.8), but wide limits of agreement and proportional bias.

This is the first study where the FACIT-F questionnaire was administered to former ICU patients. One year after hospital discharge, the mean raw FACIT-F score, which has a range 0-52, was 39.1 ± 10.1. The mean raw values reported in studies performed in different settings ranged from 29.1 [25] to 35.8 [12] in rheumatologic diseases, from 24 ± 11 in iron deficiency anaemia [16] to 23.9 ± 12.6 in anaemic cancer patients [11], and from 40.0 ± 9.8 in non-anaemic cancer patients [11] to 43.6 ± 9.4 in a sample of the general USA population [11]. Therefore, fatigue reported by our study patients was lower than that reported in studies performed in patients, and more similar to that of a normal USA population.

As far as validation of FACIT-F is concerned, Cronbach’s α of 0.937 for our patients was high, and similar to that reported by others [12, 13, 16]. This finding indicates good reliability of the questionnaire, whose questions are likely to measure the same construct. Moreover, the FACIT-F values did not show a floor or ceiling effect (data not shown). We also tested the construct validity considering that the grade of the MRCD scale should be related to fatigue, and we found that FACIT-F was significantly related to the MRCD scale. Therefore, we can conclude that the FACIT-F questionnaire is valid for ICU survivors assessed at one year.

The secondary aim of our study was to verify whether the VT domain of SF-36, which is widely used in the assessment of ICU patients at follow-up, could substitute the most specific FACIT-F questionnaire. We found a good correlation between FACIT-F-trans (range 0-100), and VT of SF-36 (rho 0.660, p < 0.001). The Bland Altman plot showed a bias of -0.8 with 95 % limits of agreement from -34.1 to 35.7. These wide limits of agreement, and the significant proportional bias found by the linear regression suggest that VT does not allow an accurate assessment of fatigue. The reason for this discrepancy between correlation and agreement of FACIT-F and VT may depend on the items assessed by each questionnaire. The FACIT-F consists of 13 items, assessing 11 negative and 2 positive aspects (An5 ‘I have energy’, and An7 ‘I am able to do my usual activities’), while the VT of SF-36 consists of 4 items, that assess 2 positive and 2 negative aspects. We can hypothesize that FACIT-F may grasp the negative aspects of fatigue better than VT.

The VT of SF-36 in our patients was clinically better than that of the adjusted Italian normal population [24]. In detail, values clinically worse (mean of study patients minus normative mean > 5) than that of the adjusted Italian normal population were found for RP, while clinically better values were found for BP, GH, and MH dimensions (Table 2). Many studies assessing quality of life at one year by SF-36, in mixed ICU patients of different countries, generally found values of SF-36 dimensions clinically lower than that of the adjusted normal population [26–31]. Hence, our patients experienced a relatively good quality of life one year after hospital discharge, comparable to that of the adjusted Italian normal population. We do not have an explanation for this finding. Nevertheless, Chiumello et al. [32], who evaluated 26 Italian ARDS patients ventilated in supine or prone position at the one-year stage, had similar results. Interestingly, the assessments of both our patients and those of Chiumello et al. [32] were performed by direct interview at the hospital, and the percentages of the eligible patients assessed were 43 % and 39 %, respectively. We excluded patients residing farther than 30 km from the hospital, and Chiumello et al. [32] excluded patients residing more than 40 km from the hospital. Of the 15 patients (11.5 %) who refused to participate, some claimed that they had difficulty in reaching the hospital. Therefore, the assessment of patients at the hospital may create a selection bias. An alternative hypothesis is that the Italian normative sample, collected in 1995 [24], may not be suitable for comparisons after 20 years. Indeed, the country with the world's second highest life expectancy in 2012 and 2013 [33], has a relatively healthy cuisine and diet, and a good healthcare system. Possibly, all these factors may have positively affected health-related quality of life over time, with better actual normal values than in the past.

This study has strengths and limitations. As far as the former are concerned, the comparison between the patients interviewed and those missing the interview showed no statistically significant differences, except for BMI and LOS in ICU, which were slightly different (Table 1). Therefore, our findings should be of general value although we cannot rule out any undetected selection bias. The exclusion of patients living over 30 km from the hospital is a limitation, and we do not know whether some refusals may mask any symptoms related to Post Traumatic Stress Disorder [34]. Collecting data by direct interview allowed us to have very little missing data (33 of 2744 items, 0.01 %), and guarantees the absence of interference from family members or other persons in the answers to the questionnaires. Moreover, it allowed all patients to give their written informed consent to the study, as required by the Ethics Committee.

## Conclusions

This study validates the 13-item FACIT-F scale to assess fatigue in ICU survivors one year after hospital discharge. The Vitality dimension of SF-36 correlates with the FACIT-F scale, but consists of only four items assessing two positive and two negative aspects of vitality. FACIT-F grasps the negative aspects of fatigue better than the Vitality dimension of SF-36. Specific tools assess specific conditions better that general tools. This information should be useful for future studies designed to investigate fatigue in former ICU patients.

## Abbreviations

ARDS: Acute respiratory distress syndrome
BMI: Body mass index
BP: Bodily pain
COPD: Chronic obstructive pulmonary disease
FACIT-F: Functional assessment of chronic illness therapy fatigue
GH: General health
ICU: Intensive care unit
MH: Mental health
MRCD: Medical research council dyspnoea
PF: Physical functioning
RE: Role limitation due to emotional problems
RP: Role limitation due to physical problems
SAPS: Simplified acute physiology score
SF: Social functioning
SF-36: 36 items short-form health survey
SOFA: Sequential organ failure assessment
VT: Vitality domain
